# Thinking Like a Duck: Fall Lake Use and Movement Patterns of Juvenile Ring-Necked Ducks before Migration

**DOI:** 10.1371/journal.pone.0088597

**Published:** 2014-02-14

**Authors:** Charlotte L. Roy, John Fieberg, Christopher Scharenbroich, Christine M. Herwig

**Affiliations:** 1 Wetland Wildlife Populations and Research Group, Minnesota Department of Natural Resources, Bemidji, Minnesota, United States of America; 2 Department of Fisheries, Wildlife and Conservation Biology, University of Minnesota, St. Paul, Minnesota, United States of America; 3 Minnesota Department of Natural Resources, Bemidji, Minnesota, United States of America; Hungarian Academy of Sciences, Hungary

## Abstract

The post-fledging period is one of the least studied portions of the annual cycle in waterfowl. Yet, recruitment into the breeding population requires that young birds have sufficient resources to survive this period. We used radio-telemetry and generalized estimating equations to examine support for four hypotheses regarding the drivers of landscape scale habitat use and movements made by juvenile ring-necked ducks between the pre-fledging period and departure for migration. Our response variables included the probability of movement, distances moved, and use of different lake types: brood-rearing lakes, staging lakes, and lakes with low potential for disturbance. Birds increased their use of staging areas and lakes with low potential for disturbance (i.e., without houses or boat accesses, >100 m from roads, or big lakes with areas where birds could sit undisturbed) throughout the fall, but these changes began before the start of the hunting season and their trajectory was not changed by the onset of hunting. Males and females moved similar distances and had similar probabilities of movements each week. However, females were more likely than males to use brood-rearing lakes later in the fall. Our findings suggest juvenile ring-necked ducks require different lake types throughout the fall, and managing solely for breeding habitat will be insufficient for meeting needs during the post-fledging period. Maintaining areas with low potential for disturbance and areas suitable for staging will ensure that ring-necked ducks have access to habitat throughout the fall.

## Introduction

Managing wildlife to achieve population abundance goals requires sufficient resources for all stages of the annual cycle. The post-fledging period is an important link between the brood-rearing period and recruitment into the breeding population, but remains one of the least studied periods [1]–[3]. Recognition of this information gap has resulted in a proliferation of studies of post-fledging ecology in the last decade, but studies of post-fledging waterfowl remain relatively sparse [4]–[11].

Importantly, habitat requirements may differ for each life-history stage (e.g., post-fledging, breeding, and post-breeding birds) [4], [12]. Differences in habitat use and requirements of juveniles and adults could be due to dissimilarities in diet [13]–[15], avoidance of conflict with conspecific adults [4], [16], the need of young birds to become familiar with the landscape compared to prior knowledge of adults, and differences in the timing of migration [17] and molting chronology [17]–[18]. Differences in habitat use are evident between breeding black ducks (*Anas rubripes*), which use forested wetland and scrub-shrub habitats, and post-fledging black ducks, which use palustrine emergent and riverine habitats [4]. Like many other species of waterfowl [19], female ring-necked ducks (*Aythya collaris*) usually leave broods to molt and may undergo a molt migration [17]–[18]. Similarly, adult male ring-necked ducks depart for wintering areas before adult females and juveniles [17]. Differences in the needs of young and adults may even result in trade-offs between offspring survival and future fecundity of adult females [10], [20].

We expected habitat use and movements of newly flighted birds to be dynamic during the fall because they are exploring the landscape for the first time. Few studies have examined movements of post-fledging birds in the context of exploration and preparation for migration [1], [3], [12], [21]–[22], but see [7] and [9], in part because the high mobility and mortality of birds during this period poses challenges to data collection. We expected resource requirements to change during this period because of physiological changes associated with preparation for migration [23]. Concurrently, young ducks experience hunting pressure for the first time, which might displace birds from preferred foraging areas [24]–[26], render some habitats unavailable during intense disturbance [27]–[28], and redistribute birds both locally and regionally [29]–[31]. Young birds might also be more vulnerable to hunting than other cohorts [32].

We explored changes in habitat use and movement patterns of birds between the pre-fledging period and departure for migration during the fall. We considered four possible, but non-mutually exclusive drivers of post-fledging habitat use and movements. Our goal was to evaluate support, or lack thereof, for three hypotheses from the literature (with some modifications for application to waterfowl) and a fourth hypothesis we called the hunting- disturbance hypothesis (Table 1).

**Table 1:** Hypotheses we considered to explain movements and lake use of juvenile ring-necked ducks in the fall.

Hypothesis	Explanation	Predictions
Habitat-optimization	*Birds leave brood-rearing lakes to locate habitat that meets* *changing resource needs*	-Birds do not return to brood-rearing lakes because they no longer meet resource requirements.
		-Birds make extensive use of new lakes with adequate resources.
Future-breeding site selection(territory selection)	*Females prospect for future breeding sites during the fall, and* *thus their movements differ from those of males*	-Habitat use and movements of males and females differ.
		-Female movements are more localized to areas from which they fledged.
		-Females are more likely to return to brood-rearing areas than males.
Staging/navigational targetformation	*Bird movements and lake use are driven by preparation for* *migration*	-Movements orient along an E-W axis to form a large navigational target.
		-Birds make extensive use of staging areas after locating them.
		-Birds return to brood-rearing lakes only if they are staging areas.
		-Southerly oriented movements indicate initial migratory displacements.
Hunting-disturbance	*Movements and lake use are heavily influenced by hunting* *disturbance in the fall*	-The proportion of birds moving increases with the onset of hunting.
		-The distances birds move increases during hunting relative to preseason.
		-Birds shift to areas that are less accessible to hunters.
		-Effects are most pronounced during opening week when hunting effort is greatest.

These hypotheses are not mutually exclusive.

The habitat-optimization hypothesis has been examined by numerous studies of passerines [3], [12], [22]. This hypothesis suggests foraging requirements, predator avoidance, or physiological constraints drive selection of new habitats by post-fledging birds [21], [33]–[34]. Important predators of juvenile ring-necked ducks in Minnesota include raptors and mink [18], but little data are available for adults outside the nesting season. If ring-necked ducks have habitat requirements after fledging that cannot be met on natal or brood-rearing lakes, then birds should be expected to leave these lakes and not return. Upon finding an area with suitable resources, birds would be expected to limit movements and make extensive use of the new location.

The future breeding-site selection or territory-selection hypothesis [1], [3], [12], [22] was originally conceived for male passerines [21], [35]. In waterfowl, unlike most other birds, females are philopatric and males disperse [36]. This hypothesis can be easily modified to apply to waterfowl, as its main premise is that exploratory movements in the fall serve to identify future nesting areas. In wood ducks, yearling females that dispersed from their natal areas before migration, later returned to these more distant areas (<130 km) to breed, whereas, females that remained on their natal areas during fall later nested on their natal areas [37]. In contrast, males return to the natal areas of their mates the following spring. Male diving ducks usually do not return to their natal areas to breed [38].

To support the future-breeding site selection hypothesis, we would expect differences in the habitat use and movement rates of males and females, with females making more use of areas with breeding habitat than males. Females might also make more localized movements, staying in closer proximity to brood-rearing areas where they fledged successfully [1], or even returning to brood-rearing lakes. Males might also be expected to migrate before females (e.g., either at an earlier age or date) if females benefit from additional time prospecting or learning the landscape. Environmental cues help coordinate and synchronize conspecific departure for migration [23], but age and sex might also be important influences on staging behavior and the timing of departure [17].

The staging hypothesis, which has various names and versions in the literature [3], [12], [22], contends that post-fledging movements serve to initiate or prepare for migration, or gather navigational information for the return. Birds might familiarize themselves with numerous areas to create a “navigational target” that they would be more likely to recognize upon return from migration [1], [39]–[40]. Movements oriented along an east-west axis (perpendicular to the axis of migration) would increase the likelihood birds encounter recognizable landmarks, as opposed to movements parallel to the axis of migration which would be more easily missed [1], [3]. Staging might also have a socialization function [12], [21]. If young ring-necked ducks leave brood-rearing lakes to stage with conspecifics, then we would expect use of staging areas to increase during the fall, with a return to brood-rearing lakes only if they later become important staging areas. The exact timing of the peak in staging activity might vary among years if weather and environmental cues such as food supply are important [41]. Or, if more influenced by photoperiod [42], the timing may be fairly consistent among years. In ring-necked ducks, a bird’s internal clock is thought to be more important than weather in determining migration onset [18]. Post-fledging exploratory movements might also constitute initial migratory displacements [43], which would be expected to be oriented to the south.

The hunting-disturbance hypothesis asserts that hunting influences movements and habitat use of birds. Under this hypothesis, the proportion of birds changing locations should increase with the onset of hunting. We also expected hunting to increase the distances moved between successive locations as birds attempted to find areas free of disturbance and may have been subjected to repeated disturbances, pushing them farther than they might have otherwise moved, and potentially impairing fat acquisition and nutrient reserves [44]. A change in habitat use was expected toward 1) large lakes with areas available to escape hunters (open water hunting was not permitted during this study), and 2) lakes with no boat access, no houses, or greater distance from roads, which we assumed would reduce accessibility to hunters and hunting pressure. We expected this effect to be greatest the week following the opening of hunting season because this is when hunting pressure was greatest [45].

**Figure 1:**
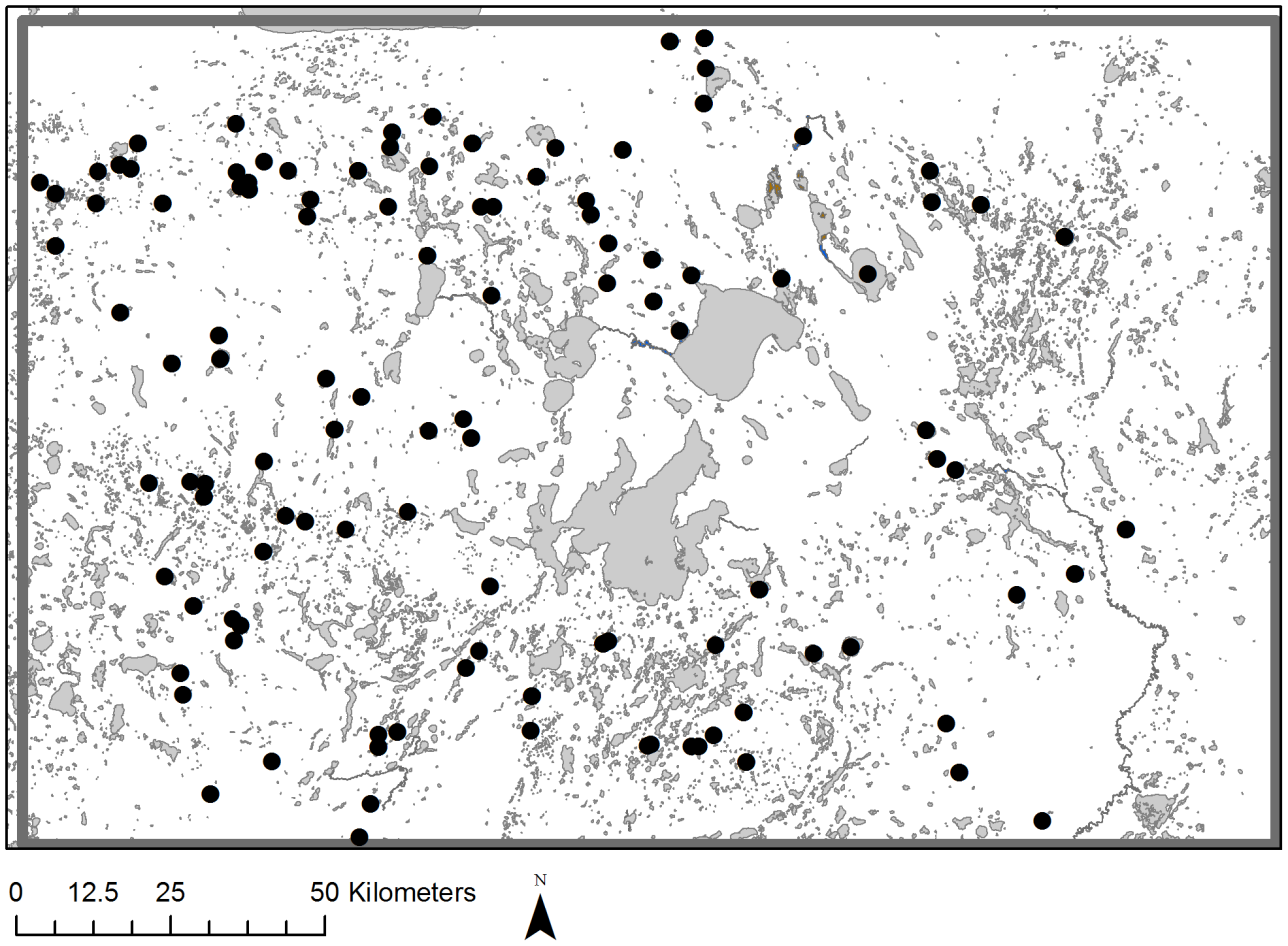
Study area and capture locations of young ring-necked ducks. Black dots indicate capture locations of birds on brood-rearing lakes in north-central Minnesota, USA during 2007–2010. Lakes and rivers are depicted in grey.

This study contributes to our understanding of post-fledging movements and habitat use by using aerial telemetry to locate birds that can move 125 km or more between locations each week. An additional strength of this study is that the study area contained an extremely large number of lakes and rivers (1000’s, Fig. 1) which provided an opportunity to examine variability in lake choices made by birds in the fall.

### Study Area

The study area was 200 km×130 km in north-central Minnesota, USA (Fig. 1) and was dominated by mixed forests and wetlands. The majority of wetlands were shallow open water systems, but deep marshes, peatlands, and bogs were also common. Hereafter, we refer to all wetlands generically as lakes. Portions of five Ecological Classification Sections [46], http://www.dnr.state.mn.us/ecs/index.html Accessed May 31 2013) were included; Lake Agassiz/Aspen Parklands, Northern Superior Uplands, Northern Minnesota and Ontario Peatlands, Northern Minnesota Drift and Lake Plains, and Minnesota and Northeastern Iowa Morainal, but the majority of locations occurred within the latter 2 sections.

## Methods

### Duckling Capture and Transmitter Implantation

United States Geological Survey permits were issued to J. Berdeen (# 05838) for trapping and marking ring-necked ducks. We captured 52 (46∶54 male:female percentage), 56 (68∶32), 68 (49∶51), and 64 (52∶48) ducklings during 4 August–3 September 2007, 29 July–26 August 2008, 27 July–25 August 2009, and 29 July–20 August 2010, respectively, using nightlighting techniques from motorized square-sterned canoes [47]. We limited the number of ducklings (≤ 2) obtained from the same lake each year to reduce non-independence that might result from correlations among brood-mates, lake or maternal effects. Captured ducklings were sexed by cloacal eversion, weighed to the nearest 5 g, and aged according to methods described in [48].

Transmitters (ATS, Isanti, Minnesota, USA) were surgically implanted beneath the skin according to methods described in [49] and used successfully on waterfowl and waterbirds [50]. We applied mesh backing to the transmitter with aquarium-grade silicon to increase transmitter retention rates (D. M. Mulcahy, U.S. Geological Survey Alaska Science Center**,** personal communication). Each transmitter weighed ∼11 g (with mesh). Transmitter-implantation surgery was completed in ∼15 minutes while birds were under the anesthetic isoflurane (4.0-5.0%, Isoflurane USP, Abbott Laboratories, Chicago, Illinois, USA). We made a ∼30 mm incision on the upper back, created a subcutaneous pocket with hemostats for the transmitter and a small hole for the 25 cm antenna to exit, and sutured the incision. We applied a topical antibiotic to the incision and antenna exit site and injected each bird intramuscularly with penicillin (0.25 ml of 1∶3 dilution of 300000 Units/ml) to prevent infection. Each bird was allowed to recover for several hours after surgery before being returned to their capture lake. In 2007 only, we placed nasal saddles on birds as part of a pilot study to examine philopatry to natal areas, but we lacked sufficient resources to resight birds the following year, and stopped.

Transmitters had a range of <2 km from the ground and ∼8 km from the air. Until birds could fly, we were able to track ducklings from the ground. When birds became flighted, we used a Cessna aircraft (Cessna Aircraft Company, Wichita, Kansas, USA) with two mounted 4–element Yagi antennas to locate birds each week during the day (0800–1630 hours). We generally spent >10 hr/week flying, but the duration of flights was dependent on weather. We tracked birds until 8 Nov 2007, 18 Nov 2008, 9 Nov 2009, and 8 Nov 2010.

### Habitat Variables

For each lake at which a bird was located, we determined area and human disturbance or access variables using GIS data layers from ftp://gdrs.dnr.state.m.us/gdrs, with data development accomplished using ArcGIS 10.0 [51]. We used the Public Water Inventory (PWI) Basin Delineations layer to obtain lake area. When a wetland, lake, or river area was not delineated as a basin in the PWI layer, we digitized the ordinary high water mark to determine area. Variables related to human disturbance or access included public boat accesses (walk-in or trailer), houses with docks, and distance between the wetland edge and nearest road. We used the Water Access Sites in Minnesota GIS data layer to identify public boat accesses at each lake. Houses with docks were determined from the Bing Aerial Photography layer (2012 Microsoft Corporation, Available Exclusively by DigitalGlobe, Imagery Date May 2011). The location of roads was determined from the Minnesota Department of Transportation Basemap Roads –All Types GIS layer. The distance between roads and the wetland edge was measured from 2010 Farm Service Agency color aerial photography from the Minnesota Geospatial Image Service (http://www.mngeo.state.mn.us/chouse/wms/wms_image_server_specs.html). Waterfowl Refuge status was determined from Minnesota Hunting Regulations Handbooks (http://files.dnr.state.mn.us/rlp/regulations/hunting/2010/waterfowl.pdf, Accessed 20 March 2013) and defined as areas closed to public hunting. A lake was identified as a staging area based on observations of 100’s to 1000’s of ring-necked ducks during fall waterfowl surveys (S. Cordts, Minnesota Department of Natural Resources, pers. comm.). These groups were likely composed of both migrants and local birds. Staging lakes comprised 12% of lakes where ring-necked ducks were detected.

Using these data layers, we created 5 lake-type categories used by radio-marked ring-necked ducks: 1) big (>5,000,000 m^2^; n = 30), 2) medium (500,000–5,000,000 m^2^) with high potential for disturbance (n = 82), 3) medium with low potential for disturbance (n = 24), 4) small (<500,000 m^2^) with high potential for disturbance (n = 178), and 5) small with low potential for disturbance (n = 100). Lakes near a road (<100 m from the edge of the wetland vegetation, not the edge of the water), with a boat access, or with a house were categorized as having high potential for disturbance. Big lakes were not subdivided based on disturbance, because their large size afforded some areas where the birds could sit undisturbed in the fall (i.e., natural refugia), despite the presence of one or more disturbance factors (e.g., boat accesses, houses, etc.). Thus all big lakes were considered to have a low potential for disturbance. The birds also used 19 lakes on waterfowl refuges which had low potential for disturbance; these refuge lakes were categorized as: big (n = 4), medium (n = 7), and small (n = 8).

### Statistical Analysis

We evaluated support for the different hypotheses (Table 1) governing movements and habitat use of juvenile ring-necked ducks by fitting a series of models to response variables measuring movement and lake-use characteristics (Table 2). We explored changes in movement rates by modeling a binary indicator variable equal to one if the bird was observed to have moved to a new lake between weeks *t*-1 and *t* and zero otherwise (observations were set to missing if the bird was not seen during either week *t-*1 or week *t*). In addition, we modeled distance between weekly locations (with observations set to missing if the bird stayed on the same lake between weeks or if it was not located during either week *t-*1 or week *t*); we summed distances between successive locations on the rare occasion birds were seen on two different lakes during the same week (n = 26 out of a total of 1,802 observations). We also modeled three binary indicator variables to explore changes in the propensity of birds to use certain lake types over time. Specifically, we modeled the probability of finding a bird on its brood-rearing lake, a lake with a low potential for disturbance, or a staging lake.

**Table 2:** Summary of ΔQIC* measures (model weights†) associated with generalized estimating equation (GEE) models fit to each of 2 movement-based response variables and 3 wetland characteristics response variables.

Model	P(transition to a newlake between *t*−1and *t* | given seen2 weeks in a row)	Distance moved,given the birdmoved betweenweeks *t*−1 and *t*	P(finding a bird on a‘non-disturbed lake’)‡	P(finding a bird on astaging lake)	P(finding a bird on itsbrood-rearing lake)
∼Week * Sex+Year	5.43 (0.057)	10.72 (0.004)	5.51 (0.049)	8.00 (0.012)	**0.00 (0.892)**
∼Week * Sex	10.63 (0.004)	11.66 (0.002)	9.36 (0.007)	4.07 (0.086)	4.28 (0.105)
∼Week+Year	**0.00 (0.853)**	**0.00 (0.841)**	**0.00 (0.774)**	1.93 (0.249)	11.80 (0.002)
∼Week	4.59 (0.086)	3.41 (0.153)	3.04 (0.169)	**0.00 (0.654)**	14.28 (0.001)

^*^QIC (Pan 2001) is a measure of model performance for general estimating equations; smaller values indicating better fit, and ΔQIC = QIC – min(QIC).

† Model weights = 
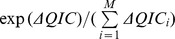
, where M = the number of models in the model set.

‡ Non-disturbed lakes included all lakes >5,000,000 m^2^ in size, waterfowl refuges, and lakes >100 m from a road with no boat access or house on the lake.

Bold values indicate the ‘best fit’ model for each response variable.

In each case, we fit a series of four nested regression models in which we modeled changes in the response as a function of: 1) observation week (week 1 = last week of July, first week of August; week 16 = 2^nd^ week in November); 2) observation week, sex, and the interaction between observation week and sex; 3) observation week and year; and 4) observation week, sex, year, and the interaction between observation week and sex. We used a set of natural cubic regression spline basis functions with 4 degrees of freedom [52] to flexibly model non-linear seasonal changes in response patterns. We modeled year effects using 3 dummy indicator variables. To account for repeated measures on each bird (and potential within-individual correlation patterns), we used a generalized estimating equations (GEE) approach [53]–[54], with models fitted using the geepack package in program R [55]–[56]. GEEs are a natural extension of generalized linear models (GLMs) to repeated measures data. Like GLMs, GEEs allow one to incorporate mean-variance relationships associated with common probability distributions traditionally used to model non-Gaussian data. For example, binary data are often assumed to follow a Bernoulli distribution in which Var[*Y | X*] = E[*Y | X*](1−E[*Y | X*]). Similarly, count data are often assumed to follow a Poisson distribution in which Var[*Y | X*] = E[*Y | X*]. When fitting GLMs, observations are effectively weighted by 1/Var[*Y | X*]. GEEs are constructed similarly, but observation weights also depend on a working correlation assumption that describes the dependence among observations from the same sampling unit (individual ring-necked ducks in this case). Robust standard errors for model parameters are constructed by considering the variability among sampling units rather than individual observations. Importantly, estimates of model parameters will be asymptotically unbiased even if the working correlation is misspecified, although more appropriate assumptions regarding the correlation structure should lead to more efficient estimates [57]. Multiple working correlation structures can be examined as part of a sensitivity analysis.

We specified a first-order autoregressive working correlation structure when fitting GEEs. We also fit models with independence and exchangeable working correlation assumptions as part of a sensitivity analysis. We compared the fit of models using QIC [58], making use of the ‘model.sel’ function in the MuMin R library [59]. In addition, we plotted seasonal trends in empirical and ‘best-fit’ model-based estimates of E[*Y | X*] to examine goodness-of-fit and to assess biological significance of the results.

## Results

**Figure 2:**
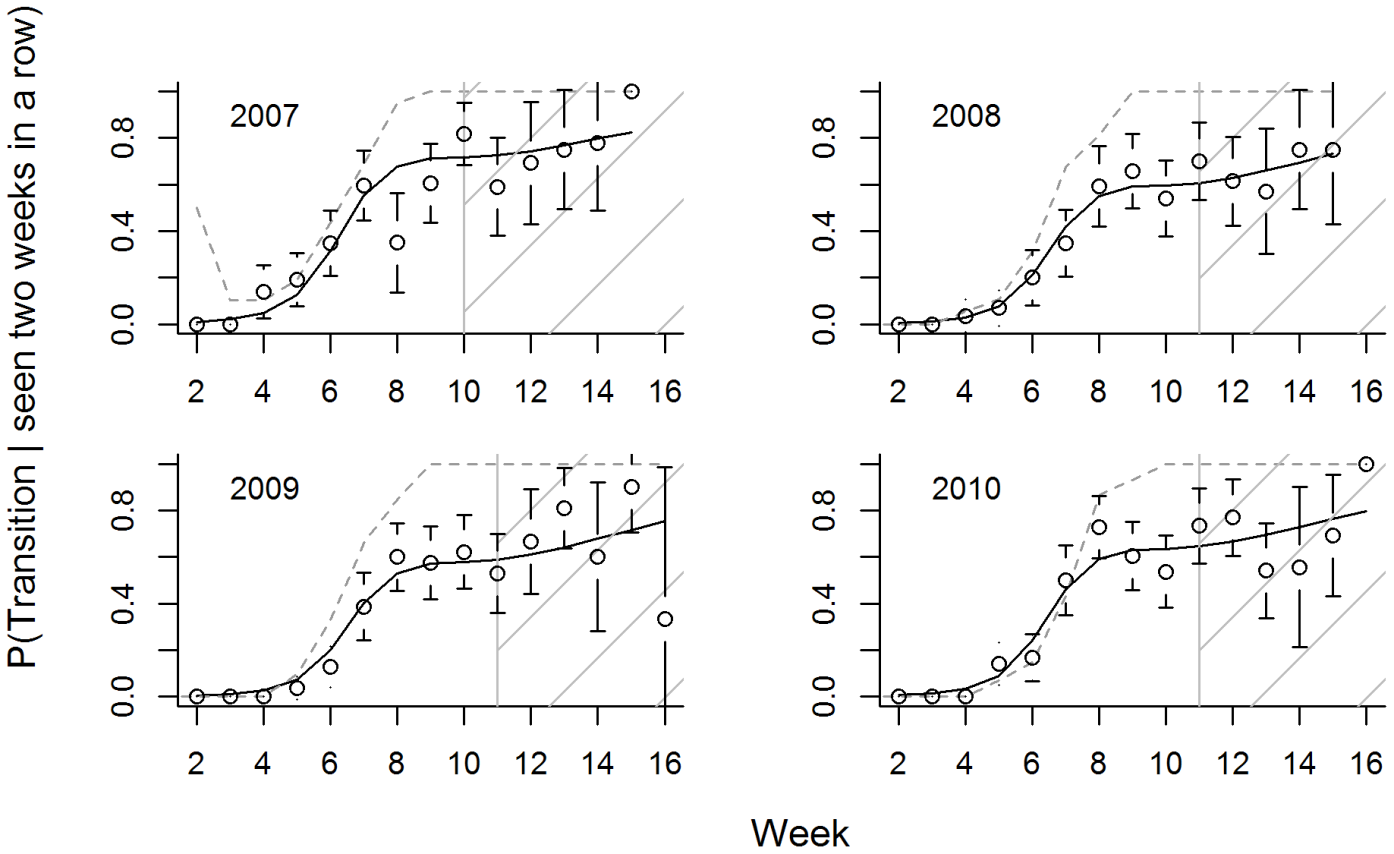
Probabilities that juvenile ring-necked ducks changed lakes, given observation in 2 successive weeks. Open circles correspond to observed proportions (with 95% confidence intervals). Solid black line gives predictions from the ‘best-fit’ generalized estimating equation model (chosen using QIC). The gray dotted line represents the proportion of marked birds flighted each week in the falls of 2007–2010. The gray vertical line (and hatched fill) demarcates the hunting season.

**Figure 3:**
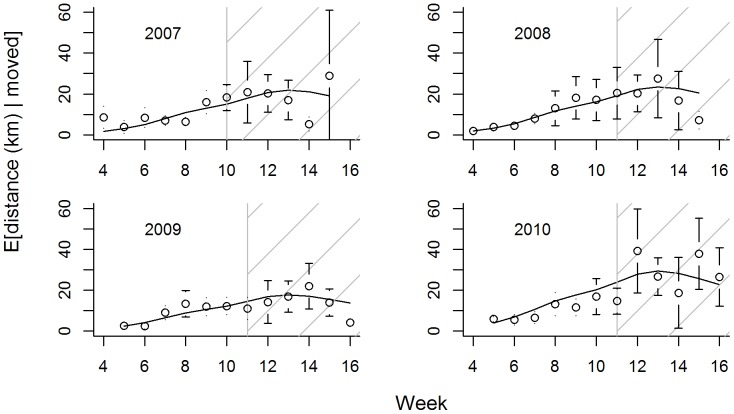
Distances radio-marked ring-necked ducks moved between locations in successive weeks, given they changed lakes. Open circles correspond to observed proportions (with 95% confidence intervals) during the falls of 2007–2010. Solid black line gives predictions from the ‘best-fit’ generalized estimating equation model (chosen using QIC). The gray vertical line (and hatched fill) demarcates the hunting season.

The proportion of birds moving to new lakes increased with observation week (Fig. 2), and birds moved greater distances, on average, when they did move (Fig. 3). Increases in transition rates (i.e., movement between lakes) generally followed the same seasonal pattern in each year, with increasing trends coinciding largely with trends in the proportion of flighted birds (Fig. 2). Importantly, movement rates stabilized around week 7 or 8 (early September), and we did not observe significant changes in movement characteristics (transition rates, average distance moved) following the onset of hunting (week 10 in 2007, week 11 in 2008–2010; Figs 2, 3). Models with observation week and year provided the best fit to both movement responses (Table 2) and results were robust to the choice of working correlation structure. Thus, we did not detect significant differences among males and females in their propensity to move to new lakes or in the average distance moved each week.

**Figure 4:**
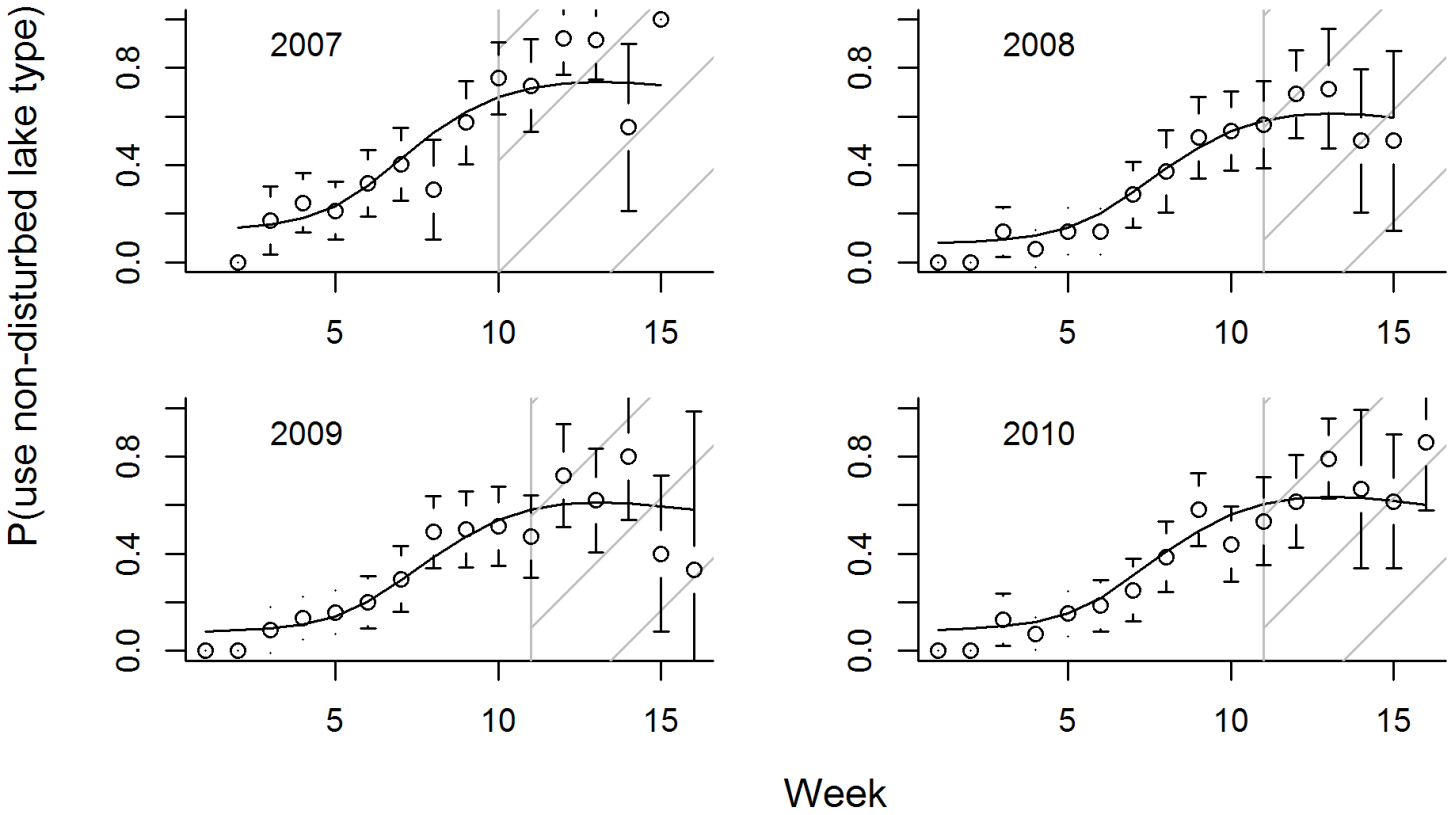
The probability that juvenile ring-necked ducks were observed on non-disturbed lakes. Non-disturbed lakes included all lakes >5,000,000 m^2^ in size, waterfowl refuges, and lakes >100 m from a road with no boat access or house. Open circles correspond to observed proportions (with 95% confidence intervals) during falls of 2007–2010. Black lines give predictions from the ‘best-fit’ generalized estimating equation model (chosen using QIC). The gray vertical line (and hatched fill) demarcates the hunting season.

**Figure 5:**
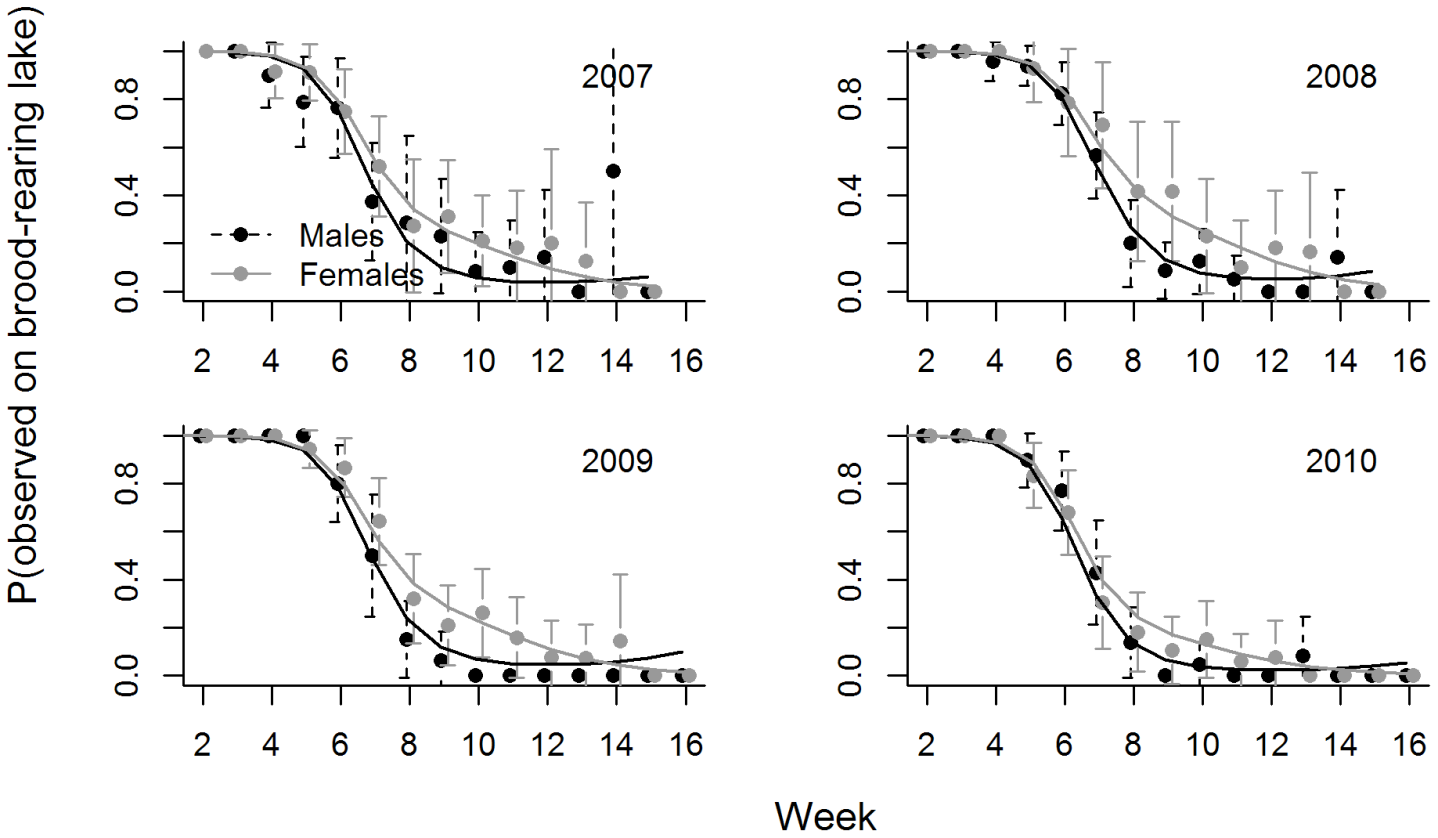
The probability that juvenile ring-necked ducks were observed on brood-rearing lakes. Open circles correspond to observed proportions (with 95% confidence intervals) during falls of 2007–2010. Black and gray lines give predictions from the ‘best-fit’ generalized estimating equation model (chosen using QIC) for males and females, respectively.

Birds also used different types of lakes as the season progressed (Fig. S1, Supporting information). In particular, ducks used bigger lakes, lakes with less potential for disturbance, and staging lakes more frequently as the season progressed (Table 2; Fig. 4, Fig. S2, Supporting information). As a corollary, they used smaller lakes less frequently and were less likely to be detected on brood-rearing lakes later in the season (Fig. 5). Yet, females were more likely to be observed on their brood-rearing lake than males as the season progressed (Table 2, Fig. 5). As with movement characteristics, seasonal patterns changed smoothly over time, patterns were largely consistent across years, and we did not detect abrupt changes in use patterns in response to the onset of hunting. Models with observation week and year provided the best fit to indicator variables representing whether birds were on lakes with a low potential for disturbance, whereas a model with observation week (only) provided the best fit to an indicator variable for finding a bird on a staging lake (Table 2). Lastly, a model with year, sex, week, and the interaction between sex and week provided the best fit to the probability of observing birds on brood-rearing lakes (Table 2).

## Discussion

Our data provided mixed support for the staging hypothesis and the future breeding-site selection hypothesis, but did not support the hunting-disturbance or the habitat optimization hypotheses. Juvenile ring-necked ducks shifted to bigger, less disturbed lakes as the season progressed, but they did not settle down at lakes used after departure from brood-rearing lakes, and occasionally returned to brood-rearing lakes. Juvenile ring-necked ducks were also more likely to use staging lakes later in the season. These changes in distributional patterns began before hunting season, suggesting the underlying cause was not hunting.

We were surprised by the general lack of support for the hunting-disturbance hypothesis. We expected to see pronounced effects of hunting on movements and habitat use during the first weeks of the hunting season, because almost half the duck harvest in Minnesota occurs the first 10 days of the season [45]. Other studies have noted immediate effects of hunting on movements [28], [60] and disturbance can cause waterfowl to abandon areas and depart early from migration staging areas [26], [60]–[62]. Our study differed from most others in that we tracked the movements and habitat use of individuals on the landscape, rather than focus on disturbance at one or a few sites, or rely on counts of birds [30], [63]–[66]. Radio-marked mallards typically returned to hunted areas within a day after jump-shooting on the wintering grounds [67]. Thus, we may have seen immediate effects of hunting with more continuous monitoring, but any effects of hunting on bird movements and lake use appeared to be muted or short-lived at the landscape scale.

Birds appeared to be able to find areas with a low potential for disturbance on our study area, as indicated by their increasing use of these lakes in the fall. Importantly, our indicators of disturbance potential (e.g., houses with docks, public boat accesses, proximity to roads) were also indicators of access for other types of non-hunting disturbance such as fishing and recreational boating, and these disturbances occurred before (and after) hunting opened. Minnesota was ranked second in the United States for number of boat registrations (809,168) in 2011, and has the highest per capita boat ownership in the country (http://files.dnr.state.mn.us/aboutdnr/budget/nr_fund/14water.pdf). Some of the largest lakes in our study may have been utilized for fishing and other recreational activities, including hunting, and thus had some disturbance. However, these activities tend to be fairly localized, and some areas are generally available on these larger lakes for the birds to sit undisturbed in the fall, which was our rationale for classification. Furthermore, open water hunting was not permitted in Minnesota during this study and hunters were required to be within natural growth of vegetation and at least partially concealed. Diving ducks can rest in open water away from shorelines and use it as a refuge [28], [60], [68]. Nevertheless, we did not measure hunting or other recreational disturbance directly in this study and so we cannot be sure that our disturbance categories classified all lakes correctly. Yet, we were able to detect shifts to lakes with less potential for disturbance even if we removed these largest lakes from the analysis altogether.

Many lakes in our study area may have functioned as “unofficial” refuges for waterfowl because of their inaccessibility. Juvenile ring-necked ducks also used official refuges more during hunting season than before the onset of hunting [11], see also [31]. Starting in week 8 or 9, observations on refuges comprised a growing share of observations on lakes characterized as having low potential for disturbance (Fig. S3, Supporting information). Yet, until week 11 or 12, the majority of low-disturbance observations were on non-refuge lakes, so the observed increase in use of low-disturbance lakes was not driven solely by an increase in refuge use. The largest concentrations of migrating waterfowl occur on large, undisturbed wetlands with both forage and roost sites [66]. Disturbance-free areas have long been recognized as having value for waterfowl in the fall [63]–[65].

The probability of using a staging lake also increased throughout the fall, and was similar among years. Staging activity did not have a discernible peak (Fig. S2, Supporting information), however, late in the fall, birds tended to have more southerly-oriented movements (Fig. S4a,b, Supporting information), which could indicate initial migratory displacements [43]. This pattern is consistent with the expectation that ring-necked ducks are calendar migrators and less influenced by weather [18], which varies among years. Importantly, our estimated probability of using a staging lake was conservative since some staging lakes may not have been recognized as such because waterfowl counts were not available for all lakes used by marked ring-necked ducks. Our findings were, however, inconsistent with development of a “navigational target” [*sensu* 1], [3]; the mean distance moved along the E–W axis was similar to that along the N–S axis (Fig. S5, Supporting information). Patterns may have been somewhat obscured in our study area because although most birds are known to winter in the Mississippi Flyway, a small proportion of birds do winter in the Atlantic Flyway (Roy et al., unpubl. data), which may have produced movements along more than one axis. However, we think this effect would be small at the scale we considered; it is more likely that the influence of finer-scale variation in habitat characteristics overwhelms any navigational component to movement. Either way, we believe most birds (males and females) had migrated by the first week of November (when we stopped tracking), because lakes were icing up, few birds were located on the study area, and harvest reports of marked birds were received from outside the study area.

Similar to the staging hypothesis, the future breeding site selection hypothesis had mixed support for influencing movements. Females used brood-rearing lakes later in the fall than males which supported this hypothesis. Yet, males and females moved to new lakes at similar rates and moved similar distances. This indicates that females were not less mobile overall, and did not have more localized movements than males. It is not clear whether female use of brood-rearing habitats later in the season was related to future breeding; this pattern may have resulted from other differences between the sexes at this time of year that we did not consider. However, our data suggest that males and females leave the study area at similar times, and we anticipate resource requirements for both sexes to be related to migratory preparation and growth at this time of year. More research is necessary to explore patterns of habitat use in the fall as it relates to breeding site selection the next spring. Prospecting by yearlings has received more attention than that of juveniles, with most studies focusing on cavity-nesters whose possible nest sites are easy to identify in the fall [69]–[70].

The ability to track movements of individual birds was a strength of our study, but we recognize the distribution of ducks among the different lake types during the first few weeks of each year was heavily influenced by our use of motorized square-sterned canoes to capture ducklings. The sample of brood-rearing lakes was likely biased towards those which could be accessed with these canoes, which were likely closer to roads and thus initially biased towards the “more disturbed” lake-type category. This may have exaggerated the patterns we observed in movement towards the less disturbed category. GEEs are also most appropriate when data are “missing completely at random” – i.e., when the probability of obtaining an observation does not depend on the response of interest [53], [57]. Although this assumption was likely reasonable for indicators of whether or not our study birds were located on brood-rearing, staging, or low-disturbance lakes, we suspect birds may have been more likely to be detected if they stayed on (or near) the same lake from week to week. If true, estimates of transition rates and movement distances would be too low. Further, estimated seasonal changes in movement parameters would be attenuated towards 0 if detection rates decreased over time. Fortunately, detection rates were generally high due to the use of aerial telemetry (e.g., close to 80%, as estimated from the fit of a multi-state model to these data) [11].

An alternative analysis approach would have been to construct a series of multi-state models with additional parameters describing survival and detection rates as a function of state (e.g., lake type). We have applied this approach elsewhere to explore movement on and off of wildlife refuges [11], but our focus here was a little different – i.e., we wanted to explore how population-level lake distribution patterns changed over time; GEEs provided a straightforward and direct method for quantifying these patterns. By contrast, multi-state models would provide *indirect* estimates of lake-distribution patterns, and would require the estimation of many more parameters, including: initial state distributions, transition rates to and from each state, as well as survival and detection parameters. Any decrease in bias resulting from correcting for detection would likely come at the expense of increased variance due to the added complexity of the fitted models. Additional assumptions are also required to fit these models in discrete time [71]. For example, models are formulated under the assumption that all individuals make at most one transition per observation interval and that the timing of this transition is constant (across individuals and intervals); another assumption is that survival only depends on where the bird was at the start of the observation interval [72]. These assumptions are much too simplistic; in reality, birds are likely visiting multiple lake types during the observation intervals. Unfortunately, our sampling frequency was inadequate for capturing these finer-scale movement patterns.

This study demonstrates young ring-necked ducks do change the types of lakes they use during the day throughout the fall, suggesting that managing only for breeding habitat will not meet the needs of post-fledging birds. Managers should provide areas suitable for staging and areas with a low potential for disturbance to meet the changing needs of waterfowl in the fall. Additionally, nighttime use may differ from daytime use [Roy et al., unpubl. data], and thus nighttime monitoring might reveal use of other lake types in the fall that are important for management consideration. Finally, this study demonstrates the importance of understanding underlying seasonal or temporal patterns when interpreting the impact of hunting or other forms of human disturbance.

## Supporting Information

Figure S1 **The proportion of radio-marked juvenile ring-necked ducks observed on 5 lake types.** Disturbance (D) or the lack of disturbance (indicated by a solid bar above the letter) was used to categorize lakes beyond size to evaluate hypotheses about habitat use and movements during the falls of 2007–2010. Occassionally, birds were seen more than once in a week; No.of observations (depicted on the right axis) provides a total observation count in each week.
(DOCX)

Figure S2 **The probability that juvenile ring-necked ducks were observed on staging lakes.** Open circles correspond to observed proportions (with 95% confidence intervals) during falls of 2007–2010. Black lines give predictions from the ‘best-fit’ generalized estimating equation model (chosen using QIC). The gray vertical line (and hatched fill) demarcates the hunting season.
(DOCX)

Figure S3 **Distribution of weekly observations among potential disturbance categories (low, high) and refuge indicators (yes, no).** Data are pooled across 2007–2010. The width of each bar reflects the sample size associated with the weekly observation interval, whereas the height reflects the relative distribution of observations within each of these intervals. These data illustrate that: a) refuges are used more frequently over time, beginning in week 7 or 8; b) non-refuge, low disturbance lakes are also used more frequently over time; and c) the latter category comprises the majority of observations in the low potential for disturbance category until week 12 or 13.
(DOCX)

Figure S4 (a) Weekly fall net displacements in the Eastern (left panel) and Southern (right panel) directions averaged across ring-necked ducks. Data were collected in north-central Minnesota and pooled across 2007–2010. Net displacements represent differences in UTM coordinates between a bird’s current location, (*X_t_*, *Y_t_*), and the centroid of a bird’s natal lake (*X*_0,_ *Y*_0_). Specifically, the left panel depicts weekly among-bird means of: (*X_t_* – *X*_0_), and the right panel depicts weekly among-bird means of: −(*Y_t_* – *Y*_0_). S4b. Average weekly fall displacements in the Eastern (left panel) and Southern (right panel) directions by ring-necked ducks. Data are pooled from 2007–2010. Displacements represent differences in UTM coordinates between a bird’s current (*X_t_*, *Y_t_*) and previous locations (*X_t_*_−1,_ *Y_t_*_−1_) in north-central Minnesota. Specifically, the left panel depicts weekly among-bird means of: (*X_t_* – *X_t_*_−1_) and the right panel depicts weekly among-bird means of: −(*Y_t_* – *Y_t_*_−1_). On the rare occasion birds were seen on two different lakes during the same week (*n = *26 out of a total of 1802 observations), we summed the displacements prior to taking the among-bird average.
(DOCX)

Figure S5
**Average weekly fall distances from brood-rearing lakes calculated along north-south (N–S) and east–west (E–W) axes by sex for ring-necked ducks.** Data are pooled from 2007–2010. Distances were calculated using UTM coordinates associated with the bird’s current location, (*X_t_*, *Y_t_*). and the centroid of the bird’s brood-rearing lake, (*X*_0_, *Y*_0_) in north-central Minnesota. Specifically, the E–W lines depict weekly among-bird means of: 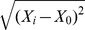, where *i* indexes successive bird locations. Similarly, the N–S lines depict weekly among-bird means of: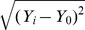.(DOCX)
