# Supplementary material for: Thinking Like a Duck: Fall Lake Use and Movement Patterns of Juvenile Ring-Necked Ducks before Migration
Source: PLoS One. 2014 Feb 14;9(2):e88597. doi: 10.1371/journal.pone.0088597 (PMC3925105; doi:10.1371/journal.pone.0088597)
Supplement: Figure S3 — Distribution of weekly observations among potential disturbance categories (low, high) and refuge indicators (yes, no). Data are pooled across 2007–2010. The width of each bar reflects the sample size associated with the weekly observation interval, whereas the height reflects the relative distribution of observations within each of these intervals. These data illustrate that: a) refuges are used more frequently over time, beginning in week 7 or 8; b) non-refuge, low disturbance lakes are also used more frequently over time; and c) the latter category comprises the majority of observations in the low potential for disturbance category until week 12 or 13. (DOCX) [file pone.0088597.s003.docx]

Figure S3.
