# Supplementary material for: Thinking Like a Duck: Fall Lake Use and Movement Patterns of Juvenile Ring-Necked Ducks before Migration
Source: PLoS One. 2014 Feb 14;9(2):e88597. doi: 10.1371/journal.pone.0088597 (PMC3925105; doi:10.1371/journal.pone.0088597)
Supplement: Figure S4 — (a) Weekly fall net displacements in the Eastern (left panel) and Southern (right panel) directions averaged across ring-necked ducks. Data were collected in north-central Minnesota and pooled across 2007–2010. Net displacements represent differences in UTM coordinates between a bird’s current location, (Xt, Yt), and the centroid of a bird’s natal lake (X 0, Y 0). Specifically, the left panel depicts weekly among-bird means of: (Xt – X 0), and the right panel depicts weekly among-bird means of: −(Yt – Y 0). S4b. Average weekly fall displacements in the Eastern (left panel) and Southern (right panel) directions by ring-necked ducks. Data are pooled from 2007–2010. Displacements represent differences in UTM coordinates between a bird’s current (Xt, Yt) and previous locations (Xt −1, Yt −1) in north-central Minnesota. Specifically, the left panel depicts weekly among-bird means of: (Xt – Xt −1) and the right panel depicts weekly among-bird means of: −(Yt – Yt −1). On the rare occasion birds were seen on two different lakes during the same week (n = 26 out of a total of 1802 observations), we summed the displacements prior to taking the among-bird average. (DOCX) [file pone.0088597.s004.docx]

Figure S4a.

**

S4b.
