# Supplementary material for: Thinking Like a Duck: Fall Lake Use and Movement Patterns of Juvenile Ring-Necked Ducks before Migration
Source: PLoS One. 2014 Feb 14;9(2):e88597. doi: 10.1371/journal.pone.0088597 (PMC3925105; doi:10.1371/journal.pone.0088597)
Supplement: Figure S5 — Average weekly fall distances from brood-rearing lakes calculated along north-south (N–S) and east–west (E–W) axes by sex for ring-necked ducks. Data are pooled from 2007–2010. Distances were calculated using UTM coordinates associated with the bird’s current location, (Xt, Yt). and the centroid of the bird’s brood-rearing lake, (X 0, Y 0) in north-central Minnesota. Specifically, the E–W lines depict weekly among-bird means of: , where i indexes successive bird locations. Similarly, the N–S lines depict weekly among-bird means of:. (DOCX) [file pone.0088597.s005.docx]

Figure S5.
